# Supplementary material for: Association of digoxin with mortality in patients with advanced chronic kidney disease: A population-based cohort study
Source: PLoS One. 2021 Jan 15;16(1):e0245620. doi: 10.1371/journal.pone.0245620 (PMC7810292; doi:10.1371/journal.pone.0245620)
Supplement: S1 Table — (DOCX) [file pone.0245620.s001.docx]

S1 Table. ICD-9-CM codes used to identify clinical conditions

| Diagnosis | Corresponding ICD-9 codes |
| --- | --- |
| Chronic kidney disease | 【585】【581.9】 |
| Acute kidney injury | 【584】 |
| Acute coronary syndrome | 【410】【411】 |
| Ischemic stroke | 【433】【434】【436】 |
| Hemorrhagic stroke | 【430】【431】【432】 |
| Diabetes mellitus | 【250】 |
| Hypertension | 【401】~【405】 |
| Hyperlipidemia | 【272】 |
| Coronary artery disease | 【410】~【414】【429.2】 |
| Cerebrovascular disease | 【430】~【438】 |
| Atrial fibrillation | 【427.3】 |
| Heart failure | 【398.91】【402.01】【402.11】【402.91】【404.01】【404.03】【404.11】【404.13】【404.91】【404.93】【428】 |
| Gout | 【274】 |
| Malignancy | 【140】~【208】 |

Footnotes: ICD, international classification of disease
