# Supplementary material for: Association of digoxin with mortality in patients with advanced chronic kidney disease: A population-based cohort study
Source: PLoS One. 2021 Jan 15;16(1):e0245620. doi: 10.1371/journal.pone.0245620 (PMC7810292; doi:10.1371/journal.pone.0245620)
Supplement: S2 Table — (DOCX) [file pone.0245620.s002.docx]

S2 Table. Drugs prescriptions during observation period among patients with chronic kidney disease

| Drug type | Drug name |
| --- | --- |
| Antiplatelets | Aspirin, Ticlopidine, Clopidogrel, |
| Warfarin | Warfarin |
| Statin | Atorvastatin,Fluvastatin, Lovastatin, Pravastatin, Rosuvastatin, Simvastatin |
| Angiotensin-converting enzyme inhibitor (ACEI) | Captopril, Enalapril, Lisinopril, Perindopril, Ramipril, Quinapril, Benazepril, Cilazapril, Fosinopril, Imidapril |
| Angiotensin receptor blockers (ARB) | Candesartan, Irbesartan, Losartan, Olmesartan, Telmisartan, Valsartan |
| Beta-Blockers | Labetalol, Pindolol, Acebutolol, Alprenolol, Atenolol, Betaxolol, Bisoprolol, Carteolol, Carvedilol, Nadolol, Metoprolol, Oxprenolol, Propranolol, Sotalol, Timolol, Metipranolol, Esmolol |
| Calcium channel blockers | Nifedipine, Nicardipine, Felodipine, Amlopidine, Isradipine, Diltiazem, Verapamil |
| Diuretics | Hydrochlorothiazide, Trichlormethiazide, Bumetanide, Furosemide |
| Oral antidiabetic agents | Acarbose, Acetohexamide, Buformin, Chlorpropamide, Gliclazide, Glimepiride, Glipizide, Gliquidone, Glyburide, Metformin, Nateglinide, Pioglitazone, Repaglinide, Rosiglitazone, Tolazamide, Tolbutamide, Digoxin, Miglitol |
| Insulin | Insulin human, Insulin zinc crystal, Insulin chromatograp, Insulin monocomponem, Insulin isophane, Insulin protamine, Insulin lispro, Insulin glargine, Insulin aspart, Insulin glulisine, Insulin detemir |
| Nonsteroidal anti-inflammatory drugs | Acemetacin, Aloclofenac, Alminoprofen, Benzydamine, Diclofenac, Diflunisal, Etodolac, Etofenamate, Fenbufen, Flufenamate, Flufenamic acid, Flurbiprofen, Ibuprofen, Iclofenac, Indomethacin, Ketoprofen, Ketorolac, Meclofenamate, Meclofenamic acid, Naproxen, Piroxicam, Spirin, Sulindac, Niflumic acid, Tenoxicam, Tiaprofenic acid, Tiaramide, Tolmetin, Tolfenamic acid, Mepirizole, Celecoxib, Meloxicam, Rofecoxib, Etoricoxib, Nimesulide |
