# Supplementary material for: Association of digoxin with mortality in patients with advanced chronic kidney disease: A population-based cohort study
Source: PLoS One. 2021 Jan 15;16(1):e0245620. doi: 10.1371/journal.pone.0245620 (PMC7810292; doi:10.1371/journal.pone.0245620)
Supplement: Attachment — Submitted filename: PONE-D-20-21787 Response to Reviewers.docx [file pone.0245620.s004.docx]

**Response to editor**

Thank the editor for the important comments. We have revised our manuscript and responded the questions as requested. Please see the response for eache question.

1. Please ensure that your manuscript meets PLOS ONE’s style requirements, including those for file naming.

Response to Q1:

Thank you for your comment. We have revised manuscript and file naming to meet PLOS ONE’s style requirements.

2. Please provide the full name of the ethics committee which approved this study in the ethics statement on the online submission form. Currently this information is only available in the methods section of your manuscript.

Response to Q2:

We have provided this information on the methods section and online submission form as “This study was approved by the Institutional Review Board (IRB) of Kaohsiung Medical University Hospital (KMUHIRB-EXEMPT(I)-20180035), and the requirement for informed consent was waived.” (page 8, line 112-115).

3. In the ethics statement in the manuscript and in the online submission form, please provide additional information about the patient records used in your retrospective study. Specifically, please ensure that you have discussed whether all data were fully anonymized before you accessed them and/or whether the IRB or ethics committee waived the requirement for informed consent. If patients provided informed written consent to have data from their medical records used in research, please include this information.

Response to Q3:

We provided this information about the patient records in the revised manuscript (page 7, line 96-101) and online submission as “We conducted a retrospective cohort study using the Pre-ESRD care program registry linked with the National Health Insurance Research Database (NHIRD), containing detailed information on inpatient and outpatient services. To protect patients’ privacy, NHIRD had made all data fully anonymized by replacing all personal identification with surrogate numbers before researchers accessed them and further analyzed them.”

Moreover, our local IRB ethics committee has waived the requirement for informed consent. This statement is stated in the revised manuscript (page 8, line 112-115) as “This study was approved by the Institutional Review Board (IRB) of Kaohsiung Medical University Hospital (KMUHIRB-EXEMPT(I)-20180035), and the requirement for informed consent was waived.”

4. Thank you for stating the following in the Competing Interests section:

“I have read the journal’s policy and the authors of this manuscript have the following competing interests: Co-author Ping-Hsun Wu is a fellow of PLOS ONE Editorial Board Members. This does not alter the authors’ adherence to PLOS ONE editorial policies and criteria.” Please confirm that this does not alter your adherence to all PLOS ONE policies on sharing data and materials, by including the following statement: “This does not alter our adherence to PLOS ONE policies on sharing data and materials.”

If there are restrictions on sharing of data and/or materials, please state these. Please note that we cannot proceed with consideration of your article until this information has been declared.

Please include your updated Competing Interests statement in your cover letter; we will change the online submission form on your behalf.

Please know it is PLOS ONE policy for corresponding authors to declare, on behalf of all authors, all potential competing interests for the purposes of transparency. PLOS defines a competing interest as anything that interferes with, or could reasonably be perceived as interfering with, the full and objective presentation, peer review, editorial decision-making, or publication of research or non-research articles submitted to one of the journals. Competing interests can be financial or non-financial, professional, or personal. Competing interests can arise in relationship to an organization or another person.

Response to Q4:

We have confirmed the competing interests in the following statement in the revived manuscript (page 21, line 359-360) as:

“Co-author Ping-Hsun Wu is a fellow of PLOS ONE Editorial Board Members. This does not alter our adherence to PLOS ONE policies on sharing data and materials.”

5. We note that you have indicated that data from this study are available upon request. PLOS only allows data to be available upon request if there are legal or ethical restrictions on sharing data publicly.

In your revised cover letter, please address the following prompts:

a) If there are ethical or legal restrictions on sharing a de-identified data set, please explain them in detail (e.g., data contain potentially identifying or sensitive patient information) and who has imposed them (e.g., an ethics committee). Please also provide contact information for a data access committee, ethics committee, or other institutional body to which data requests may be sent.

b) If there are no restrictions, please upload the minimal anonymized data set necessary to replicate your study findings as either Supporting Information files or to a stable, public repository and provide us with the relevant URLs, DOIs, or accession numbers. Please see http://www.bmj.com/content/340/bmj.c181.long for guidelines on how to de-identify and prepare clinical data for publication.

Response to Q5:

The legal and ethical restrictions in Taiwan are the main reasons for not allowing to share data publicly. We stated this explanation in the revised manuscript (page 20, line 344-357) as below. “The raw data used in our study were obtained from NHIRD and "pre-ESRD care program dataset" through formal application. The datasets we used from NHIRD included "H_NHI_OPDTE, H_NHI_IPDTE, H_NHI_DRUGE, H_NHI_OPDTO, H_NHI_IPDTO, H_NHI_DRUGO, H_NHI_ENROL, H_NHI_CATAS, and H_OST_DEATH". Data holder for NHIRD was the Health and Welfare Data Science Center, Department of Statistics, Ministry of Health and Welfare, Taiwan (<https://dep.mohw.gov.tw/DOS/np-2497-113.html>). Data holder for "Pre-ESRD care program dataset" was Information Integration and Application Center, National Health Insurance Administration, Ministry of Health and Welfare (<https://www.nhi.gov.tw/Content_List.aspx?n=2D2FAF5214807829&topn=787128DAD5F71B1A>). These raw data were limited to research purposes only and cannot be made publicly available under regulation of the "Personal Information Protection Act" in Taiwan. We, as authors, did not have any special access privileges to the data that other researchers would not have.”

**Response to Reviewer 1**

Major comments:
Q1: It would be helpful if the authors could explain in more details their statistical

plan, and the choice of the various tests they use. My understanding is that the all cause mortality outcome was assessed using a Cox proportional hazard model while the other outcomes (cardiovascular and renal) were examined using a Fine&Gray subdistribution hazard model, taking into account the competing risk of death. This should be made more clear in the tables. The tables are currently presented as if the statistical test looking at “all cause mortality” as the outcome, is also being adjusted for the competing risk of death, which is very confusing.

Response to Q1:

Thank you for the comment. Regarding the statistical plan and the choice of the various tests, we provide this information in the revised manuscript (page 11, line 161-164 ) as “Regarding the all-cause mortality outcome, we employed Cox proportional hazards model. Furthermore, we applied the Fine and Gray subdistribution hazards model to clarify the competing risk of death and the effects of digoxin on the cardiovascular and renal outcomes [21].”

We also add this information in the footnote of Table 2 to 4 as “All-cause mortality was assessed using a Cox proportional hazard model. Effects of digoxin on the cardiovascular and renal outcomes were adjusted for competing risk of mortality using a Fine & Gray subdistribution hazard model.”

Q2: How did the authors adjust for medications? Was there a code Y/N for each medication class, or were all the medications considered as one variable?

Response to Q2:

We defined each medication class as one variable with a Y/N code. We revised the sentence in page11, line 164-166, as “We applied the multivariable models to adjust the confounders for urbanization, socioeconomic status, comorbid disorders, clinical characteristics, and each medication class.”

Q3: “Poisson regression model was used to analyze the incidence rate ratios (IRRs) and 95% confidence intervals (95% CIs) of outcomes (all-cause mortality, cardiovascular events, and renal outcomes) were examined for digoxin users and non-users.” This sentence is not clear. Could the authors please explain why they chose to use a Poisson regression?

Response to Q3:

Poisson regression model can be applied to evaluate relative risk of outcomes between groups and complex interactions with covariates [1]. Compared with Cox proportional hazards model, Poisson regression model was more appropriate to evaluate relative risk of outcomes between our groups across all periods [2]. Thus, we used the Poisson regression model to estimate the effect of digoxin on the incidence rates of outcomes in this study. We revised the sentence in the revised manuscript (page 10, line 158-160) as “The incidence rate ratios (IRRs) and 95% confidence intervals (95% CIs) of outcomes (all-cause mortality, cardiovascular events, and renal outcomes) for digoxin users versus non-users were examined by using the Poisson regression model [20].”

Minor comments:

Q4: Using ICD codes to define CKD and AKI events is disputable, and using an average serum creatinine value, and the KIDGO definition of AKI may have been more accurate.

Response to Q4:

For the question about using ICD codes to define CKD and AKI events, we agree that the KIDGO definition of AKI may have been more accurate. However, previous reports also supported the rationale of using ICD codes to define AKI events. According to the study conducted by Waikar et al., ICD-9-CM codes 584 for AKI diagnosis had a sensitivity of 35.4%, specificity of 97.7%, the positive predictive value of 47.9%, and negative predictive value of 96.1% [3]. Based on this study, using the ICD-9-CM code 584 is reliable for AKI events because of high specificity although relatively low sensitivity may miss some potential candidates.

Next, for the question about using ICD codes to define CKD, we selected the CKD population for this study from pre-ESRD program registry database in addition to ICD-CM codes 585 and 581.9. The eligibility criteria for pre-ESRD program were individuals with CKD stages 3b-5 (eGFR<45 mL/min/1.73 m2), or those with proteinuria (urine protein to creatinine ratio, UPCR >1000 mg/g). The duration of the illness must be more than 3 months before enrollment to the pre-ESRD program. Thus, patients identified through these processes fit the definition of CKD by KDIGO.

Q5: “Baseline eGFR was calculated from the last recorded serum creatinine level before the index date” how long before the index date could that serum creatinine be measured ? Could an average of 2 or 3 eGFRs be used instead ? One single value for a serum creatinine (or eGFR) can be highly misleading.

Response to Q5:

For this question, we employed the enrollment criteria for entering the pre-ESRD program to explain that baseline eGFR was calculated by last recorded serum creatinine levels. The Enrollment criteria for entering the pre-ESRD program included patients with CKD stage 3b-5 or those with proteinuria >1g/day. All patients were followed up at least quarterly. The consensus for enrollment criteria was stringent for the nephrologists to select the candidate patients under NHI regulation. Thus, we considered that a single value of serum creatinine could be used as the measurement for baseline eGFR data.

**Response to Reviewer 2**

Q1: The most important shortcoming of this analysis is the fact that matching was based on age and sex only. Although they adjusted for multiple comorbidities, matching for the most relevant factors would be a better option.

Response to Q1:

We agree that adding more relevant factors in matching would make two groups more comparable in addition to age and sex matching. However, this way might reduce the sample sizes and overall statistical power. To ensure having enough patients for analysis and maintain sufficient statistical power, we aimed to match patients by age and sex for the primary analysis. Furthermore, we applied propensity scores matching to consider all covariates to increase comparability and reduce potential confounding effects between two groups. Both matching approaches consistently showed that digoxin could exert a higher risk for mortality of the study population.

Q2: In addition and most importantly, severity of HF is not properly defined. It is VERY LIKELY that more advanced HF patients were more likely to receive digoxin as recommended by the guidelines. This could explain the lower blood pressure in those taking digoxin. The authors must be very clear on this. The way how they discuss this issues is not sufficient.

Response to Q2:

In our cohort study design, the Pre-ESRD program database collected the data mainly related to renal care. Severity of HF was not available in this database. However, previous study showed that diuretics use could be a proxy for reflecting the severity of HF [4]. Thus, we adjusted confounding effect of severity of HF by the variable of diuretics use. In this way, the influence of the severity of HF on the outcomes could be attenuated after adjusting diuretics use. We admit that we cannot collect and well control all confounders due to inherent limitations of cohort study design. We have addressed these limitations in the Discussion.

The revision paragraph was shown in the limitations section of Discussion (page

18, line 306-308) as “Furthermore, cardiac functional status by NYHA classification and structural parameters by ejection fraction were unavailable for us to evaluate their impacts on the outcomes.”

Q3: Also, the authors do not provide any evidence for their Discussion about potential survivors of digoxin being included in the study. They do not have any information on this (in fact if anything can be taken from their data, it would be the opposite as longer-term treatment was numerically associated with higher risk). The authors provide a reference that new digoxin users might have a higher mortality as compared to chronic users, but this was in AF only.

Response to Q3:

We admit that this study lacked the information about digoxin chronic users or new users due to limitations of the cohort database. We agree with your concern that our previous discussion about this issue might not be convincing. Thus, we decide to delete the following paragraph in the discussion as “On the other hand, chronic digoxin users might not be as vulnerable because they might have already survived the potential harm imposed by this drug. Otherwise, they would have died, or digoxin would have been discontinued. It has been shown that in AF patients, new digoxin users were associated with higher mortality compared with non-users, while chronic digoxin users were not” (p17 in the previous manuscript).

Q4: The authors should stress even more that SDC were not available. This is crucial as effects on mortality highly depends on SDC (as cited by the authors).A substitute could be the dosage of digoxin in relation to renal function and body weight. If dosage is available the authors should provide such calculation. If not, they need to report this as additional shortcoming.

Response to Q4:

It was reported that serum digoxin concentration (SDC) could be affected by many factors, such as renal function, the bioavailability of the digoxin formulation used, the volume of distribution, the amount of extrarenal clearance, body weight, and serum albumin concentration [5]. Although some equations have been proposed to predict the SDC, their validity in Asians or advanced CKD patients was still limited [6] [7]. Thus, there might be concerns to calculate SDC by these equations in our study population. We have stated the lack of SDC as a limitation in the Discussion (Page 18, line 301-304) as “For instance, serum digoxin concentrations (SDC) were noted measured. Previous studies reported that high SDC (>1.2 ng/mL) might be associated with increased mortality when compared with low SDC (0.5–0.8 ng/mL) [36,37].”

Q5: They authors identified almost 32,000 patients but only less than 10% were actually included in the analysis. The authors report that this reduction happened after the matching process, which considered age and sex only. Sex and age is presumably known for all 32,000 patients. Why were only so few included? Was the reason that only 440 patients received digoxin (i.e. 1.4% of the total population)? The authors should include more patients or must report as a limitation that only a very small minority received digoxin which may limit generalisability of the findings. If it is possible to include more patients, it would be interesting to see results separately for patients with HF and AF.

Response to Q5:

The small sample size could be explained by that only a small portion of CKD patients met the indication for digoxin, rather than by matching process. Two reasons may help explain this phenomenon. First, guidelines do not recommend digoxin as the first-line medication for HF, and digoxin was used mainly for symptomatic HF patients despite receiving standard therapy. Second, the information about the safety of digoxin in advanced CKD patients was limited. We stated this issue in the limitation section in the Discussion (page 18, line 309-313) as below. “Another limitation was the relatively small sample sizes of digoxin users. Our study aimed to enroll more digoxin users by defining digoxin users as any single digoxin exposure within three months before the index date, but only 440 digoxin users were enrolled in total. It might be explained by the stringent indication or safety concern on prescribing digoxin for CKD patients.”

Q6: Where there also patients without HF and AF but receiving digoxin? If yes, what was the indication in those? Generally speaking, the authors should provide information on the indication for the use of digoxin in their cohort.

Response to Q6:

To our knowledge, AF and HF were the main indications for digoxin use. However, the indication for digoxin in each patient was not well recorded in the National Health Insurance (NHI) claim database. Some reasons related to the NHI claim database’s regulations may explain why patients without HF and AF might receive digoxin. First, only the first three diagnosis codes were recorded in the National Health Insurance claim database. Thus, the corresponding ICD codes for AF or HF might be missed because they were placed in the latter part of the diagnosis list in the medical record. Second, digoxin might be prescribed for the off-label condition, such as supraventricular tachycardia, not for HF or AF.

Q7: The authors refer to previous studies how ACS, ischemic stroke and haemorrhagic stroke were identified. However, the authors should briefly describe this as not all readers may have access to the referred studies.

Response to Q7:

We defined and identified the ACS, ischemic stroke, and hemorrhagic stroke based on the ICD-9-CM codes. The detailed information for this issue was stated in the Method section (page 9, line 121-126) as below. “ACS, ischemic stroke, and hemorrhagic stroke were defined as hospitalization for these vascular events, which were validated in previous studies [15,16]. For example, ICD-9-CM codes 433 (occlusion of cerebral arteries) and 434 (stenosis of precerebral arteries) were used to extract from NHIRD study subjects with ischemic stroke and were admitted for the specific diagnosis.”

Q8: In addition, they do not report on how death was identified and verified.

Response to Q8:

We identified and verified the outcome of death based on the evidence of patient withdrawal from the NHI claim database. It was reported to be valid to identify the death outcome using a similar approach [8]. We stated this issue in the Method section (page 8, line 120-121) as “Death was ascertained based on the evidence of patient withdrawal from the NHI claim database.”

Q9: The authors completed the follow-up already in 2012. What is the reason for this?

Response to Q9:

We state the following explanations for this question. First, there was a time lag between reporting the claims to the NHI database and its release for research purposes. At that time of our application for data analysis, the patient registry time was the year 2012. Second, it takes time to process and analyze the enormous cohort database and to validate their accuracy. We aimed to ensure the robustness of research design and comprehensiveness of statistical analysis for our work.

Q10: The authors should report in their conclusion that full adjustment was not possible, making bias of their findings likely.

Response to Q10:

We are aware that full adjustment was not possible, as you mentioned. We stated this limitation in the Discussion (page 19, lime 316-320) as below. “Despite these limitations, we try to reduce the influence of confounding factors on the outcomes by multivariable adjustment and propensity score matching and using different statistical analysis approaches. The inherent weaknesses of the population-based cohort study design may limit the generalizability of our findings. We should interpret the results with caution.”

References

1. Frome, E.L. and H. Checkoway, *Epidemiologic programs for computers and calculators. Use of Poisson regression models in estimating incidence rates and ratios.* Am J Epidemiol, 1985. **121**(2): p. 309-23.
2. Vonesh EF, Schaubel DE, Hao W, Collins AJ. *Statistical methods for comparing mortality among ESRD patients: Examples of regional/international variations*. Kidney International, Supplement. 2000 Dec 1;57(74).
3. Waikar, S.S., et al., *Validity of International Classification of Diseases, Ninth Revision, Clinical Modification Codes for Acute Renal Failure.* J Am Soc Nephrol, 2006. **17**(6): p. 1688-94.
4. Gislason GH, et al. *Persistent use of evidence-based pharmacotherapy in heart failure is associated with improved outcomes.* Circulation. 2007; 116(7):737-44.

5.	Iisalo, E., *Clinical pharmacokinetics of digoxin.* Clin Pharmacokinet, 1977. **2**(1): p. 1-16.

6.	Zhao, L., et al., *Efficiency of individual dosage of digoxin with calculated concentration.* Clin Interv Aging, 2014. **9**: p. 1205-10.

7.	Muzzarelli, S., et al., *Individual dosage of digoxin in patients with heart failure.* Qjm, 2011. **104**(4): p. 309-17.

8. Cheng C-L, Chien H-C, Lee C-H, Lin S-J, Yang Y-HK. *Validity of in-hospital mortality data among patients with acute myocardial infarction or stroke in National Health Insurance Research Database in Taiwan*. International Journal of Cardiology. 2015; 201:96-101.
