# Supplementary material for: Association of digoxin with mortality in patients with advanced chronic kidney disease: A population-based cohort study
Source: PLoS One. 2021 Jan 15;16(1):e0245620. doi: 10.1371/journal.pone.0245620 (PMC7810292; doi:10.1371/journal.pone.0245620)
Supplement: Attachment — Submitted filename: Response to Reviewers.docx [file pone.0245620.s005.docx]

Dear Editor and reviewers,

We appreciate your relevant comments from the Editor and reviewers. We have revised the manuscript based on reviewers’ comments and suggestions as below. Please kindly check the updated submission of the revised manuscript.

Kind regards

Jer-Chia Tsai, MD.

**Response to Reviewer 1**

*Q1: The authors have answered all my comments. I still believe however, that the tables lack clarity. The outcome “all-cause mortality” is analyzed with various models which all adjust for the competing risk of death (as stated in the foot note). An asterisk should be noted there, with mention that, for this outcome, models adjust for X, Y, Z,... but not for competing risk of death.*

**Response to Q1:**

Thank you for the comment. To clarify the statistical analysis for each outcome in Table 2, 3, and 4, we labeled the marks and the corresponding statistical analysis as:

“§All-cause mortality was assessed using a Cox proportional hazard model, and ¶Cardiovascular and renal outcomes were assessed using a Fine & Gray subdistribution hazard model for competing risk of mortality.”

**Response to Reviewer 2**

*Q1: The authors have improved the manuscript. However, they still lack sufficiently clear statements that important clinical data are missing. It is not only NYHA-class and ejection fraction, but they lack information about the presence of heart failure. This must be very clear in the text. This also means that full propensity score matching is not possible. They should also state in the conclusion that full matching was not possible to make very clear that the interpretation of their data must be done with caution.*

**Response to Q1:**

Thank you for the comment. For the information about the presence of heart failure (HF), Table 1 shows the rates of HF in the digoxin user and non-user groups were 51.6% and 10.8%, respectively. The definition of HF is based on ICD-9-CM codes 398, 402, 404, and 428. We admit that this study lacks the clinical data about NYHA-class and ejection fraction.

Following your suggestion, we state this limitation in the revision as: “Secondly, digoxin users might be frailer due to concomitant HF or AF compared to digoxin non-users. The presence of HF was defined based on ICD-9-CM coding; however, the severity of HF was unavailable due to the lack of NYHA classification and ejection fraction. Thus, full matching according to HF status in both groups was not likely, which may lead to biases.” (page 18, line 305-309)

Furthermore, we also state the limitation of this population-based cohort study design as: “However, the inherent weaknesses of the population-based cohort study design may limit the generalizability of our findings, and we should interpret the results with caution.” (page 19, line 318-320).

*Q2: They should also mention as a limitation not specifically the relatively small sample size, but the fact that only a very small portion of patients received digoxine. These patients are therefore likely not representative for the whole cohort.*

**Response to Q2:**

Thank you for the comment. Following your suggestion, we revise the paragraph as: “Thirdly, the final limitation should be considered is that a very small portion of patients received digoxin. These patients are, therefore, not fully representative of the whole cohort. This study aimed to enroll more digoxin users by defining digoxin users as any single digoxin exposure within three months before the index date, but only 440 digoxin users were enrolled in total. It might be explained by the stringent indication or safety concern on prescribing digoxin for CKD patients.” (page 18, line 310 to page 19, line 315)
