# Supplementary material for: Association of digoxin with mortality in patients with advanced chronic kidney disease: A population-based cohort study
Source: PLoS One. 2021 Jan 15;16(1):e0245620. doi: 10.1371/journal.pone.0245620 (PMC7810292; doi:10.1371/journal.pone.0245620)
Supplement: Attachment — Submitted filename: PONE-D-20-21787 Response to Reviewers.docx [file pone.0245620.s006.docx]

Response to Editor

Q1: As PLOS ONE does not provide proofreading, even small details need to be adequately addressed. In your revised manuscript you write: "Thirdly, the final limitation should be considered is that a very small portion of patients received digoxin." This is not best English and should be e.g. "Thirdly, the final limitation that should be considered is the very small portion of patients who received digoxin."

Response to Q1:

Thank you for the comment. We have revised the sentence as “Thirdly, the final limitation that should be considered is the very small portion of patients who received digoxin.” (Page 18, line 310-311)

Q2: In addition, you must temper your conclusions as addressed by reviewer #2. In the limitation section, you mention that the results must be interpreted with caution, but in the conclusion, you present your findings as "consistent evidence". This is simply not possible with the design of the study. You really need to temper this significantly and mainly say that digoxin should be given with caution, based on your results. You then can highlight the need for prospective testing.

Response to Q2:

Thank you for the comment. We have revised the paragraph as “In conclusion, our study demonstrated that digoxin use was associated with increased mortality in advanced CKD patients. Thus, digoxin should be prescribed with caution in this population. It warrants future prospective and randomized studies to determine the safety of digoxin in the advanced CKD patients.” (page 19, line 321-324)
